# Fabrication of Si negative electrodes for Li-ion batteries (LIBs) using cross-linked polymer binders

**DOI:** 10.1038/srep38050

**Published:** 2016-12-19

**Authors:** Suk-Yong Jang, Sien-Ho Han

**Affiliations:** 1 Graduate School of Knowledge Based Technology and Energy, Korea Polytechnic University 237 Sangidaehak-Ro (2121 Jungwang-Dong) Siheung-Si, Gyeonggi-Do 429–450, Republic of Korea; 2 Department of Chem. Eng. & Biotech., Korea Polytechnic University, 237 Sangidaehak-Ro, 2121 Jeongwang-Dong, Siheung-Si, Gyeonggi-Do 429-793, Republic of Korea

## Abstract

Currently, Si as an active material for LIBs has been attracting much attention due to its high theoretical specific capacity (3572 mAh g^−1^). However, a disadvantage when using a Si negative electrode for LIBs is the abrupt drop of its capabilities during the cycling process. Therefore, there have been a few studies of polymers such as poly(vinylidene fluoride) (PVdF), carboxymethyl cellulose (CMC), styrene butadiene rubber (SBR) and polyacrylic acid (PAA) given that the robust structure of a polymeric binder to LIBs anodes is a promising means by which to enhance the performance of high-capacity anodes. These studies essentially focused mainly on modifying of the linear-polymer component or on copolymers dissolved in solvents. Cross-linking polymers as a binder may be preferred due to their good scratch resistance, excellent chemical resistance and high levels of adhesion and resilience. However, because these types of polymers (with a rigid structure and cross-linking points) are also insoluble in general organic solvents, applying these types in this capacity is virtually impossible.

In theory, at least, a multifunctional monomer can easily cross-link by itself^1^,^2^,^3^,^4^,^5^, and our laboratory utilized pentaerythritol triacrylate (PETA), pentaerythritol tetraacrylate (PETTA) and dipentaerythritol pentaacrylate (DPEPA) multifunctional monomers containing three, four and five carbon-carbon double bonds at the backbone. The Si/carbon black (CB)/(poly)pentaerythritol triacrylate (PPETA) composite (PPETA-composite), Si/CB/(poly)pentaerythritol tetraacrylate (PPETTA) composite (PPETTA-composite) and Si/CB/(poly)dipentaerythritol pentaacrylate (PDPEPA) composite (PDPEPA-composite) were fabricated via a curing process from a Si/CB/pentaerythritol triacrylate (PETA) composite mixture (PETA-composite mixture), a Si/CB/pentaerythritol tetraacrylate (PETTA) composite mixture (PETTA-composite mixture) and a Si/CB/dipentaerythritol pentaacrylate (DPEPA) composite mixture (DPEPA-composite mixture), respectively^2^,^3^,^4^. The resulting charge (delithiation) rates were close to 4, 5 and 3 times higher than a Si/CB/PVdF composite containing the well-known PVdF binder for 15 cycles. Specifically, the discharge (lithiation) of the Si/CB/PVdF/PPETTA composite (1:2) (PVdF/PPETTA-composite (1:2)) (approximately 3013 mAh g^−1^) containing the PVdF/PPETTA (1:2) blended polymer (PVdF/PPETTA (1:2) binder) (blending ratio, 1.0/2.0) as a binder was improved by approximately 654 mAh g^−1^ compared to the Si/CB/PVdF composite (PVdF-composite) (about 2359 mAh g^−1^). The purpose of this study was to determine whether a relationship exists between the application of a cross-linking polymer binder and the performance of Si negative electrodes for LIBs. Another objective was to present a novel process by which to fabricate Si negative electrodes for LIBs with a cross-linked polymer binder system. Figure 1 shows the cross-linking routes of the PETA, PETTA and DPEPA multifunctional monomers.

## Results and Discussion

### Fabrication of composite electrodes

**Figure 2:**
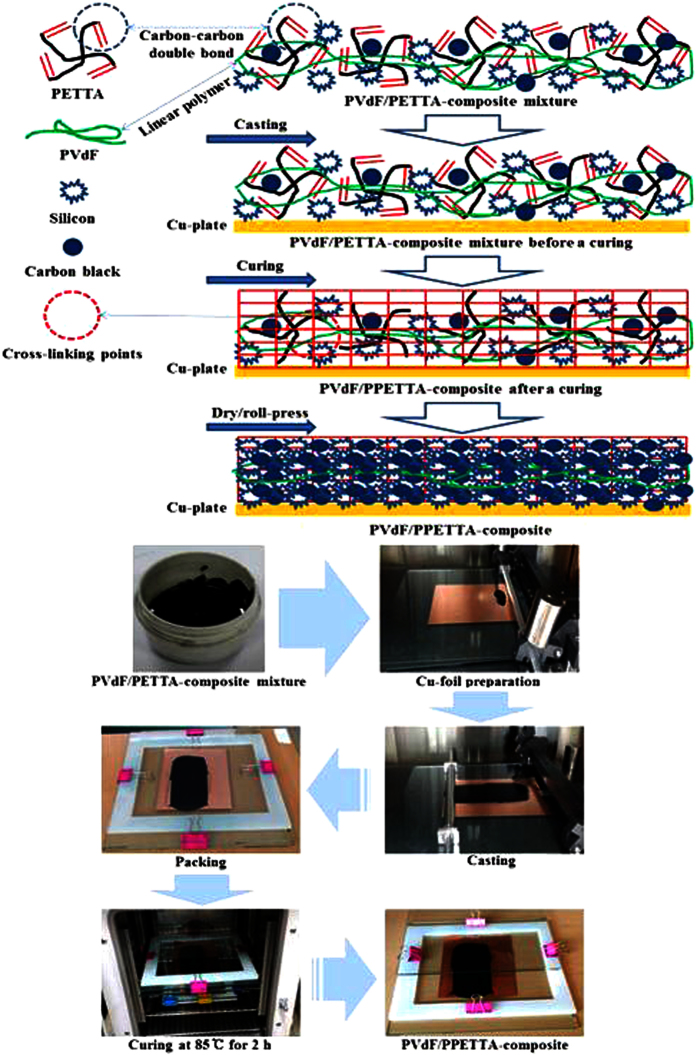
Fabrication process of the PVdF/PPETTA-composites.

**Figure 3:**
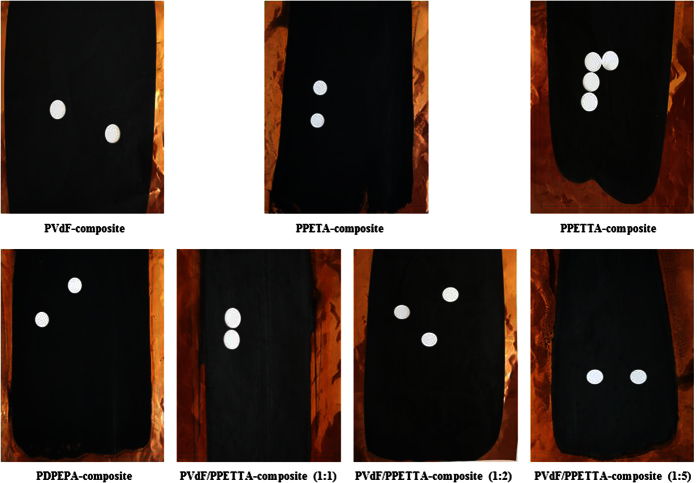
Photographs of the PVdF-composite, PPETA-composite, PPETTA-composite, PDPEPA-composite and PVdF/PPETTA-composites.

**Figure 4:**
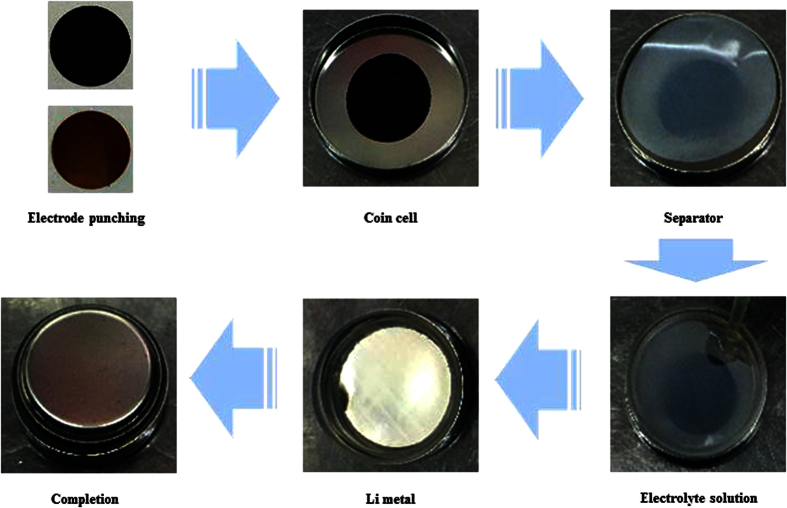
Cell production process used to create the composite electrodes.

**Figure 5:**
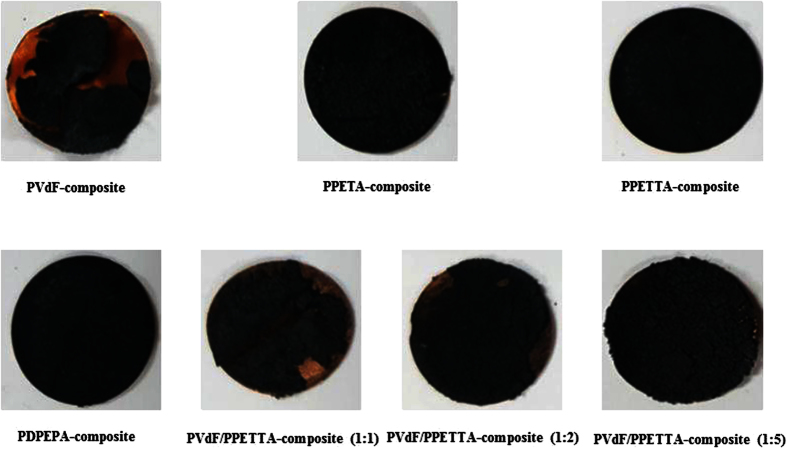
Images of the PVdF-composite, PPETA-composite, PPETTA-composite, PDPEPA-composite and PVdF/PPETTA-composites after 10 cycles.

First, the PETA-composite mixture, the PETTA-composite mixture, the DPEPA-composite mixture and the PVdF/PETTA-composite mixtures were all fabricated by the direct mixing of 60% Si, 25% CB, 15% of a binder and 2,20-azobisiso-butyronitrile (a radical initiator, AIBN) in N-methyl-2-pyrrolidone (NMP). Secondly, the composite mixtures were cast on a Cu-foil, and then cured in a silicon-packed mold for polymerization at 85 °C for 2 h^3^,^4^. Finally, the resulting composite electrodes were dried in a vacuum oven at 120 °C for 1 h. In order to enhance the inter-particle contact, the composite electrodes were roll-pressed. The entire fabrication process to create the PVdF/PPETTA-composites is shown in Fig. 2. The materials used for the fabrication of the PVdF-composite, PPETA-composite, PPETTA-composite, PDPEPA-composite and PVdF/PPETTA-composites are summarized in Table 1. Photographs of the PVdF-composite, PPETA-composite, PPETTA-composite, PDPEPA-composite and PVdF/PPETTA-composites are shown in Fig. 3. The cell production process used to create the composite electrodes is shown in Fig. 4. Images of the PVdF-composite, PPETA-composite, PPETTA-composite, PDPEPA-composite and PVdF/PPETTA-composites after 10 cycles are shown in Fig. 5.

### Fabrication of raw-binder samples

The PETA, PETTA, DPEPA, PVdF/PETTA (1:1) mixture, PVdF/PETTA (1:2) mixture and PVdF/PETTA (1:5) mixture were dissolved in NMP to form the homogeneous solutions described in Table 2. Then, a radical initiator, AIBN, was added to the solutions and directly poured into a silicon-packed mold (size: 3.0 × 3.0 cm) to carry out cross-linking polymerization for 2 h at 85 °C. After the polymerization step was complete, raw PPETA, PPETTA, PDPEPA, PVdF/PPETTA (1:1), PVdF/PPETTA (1:2) and PVdF/PPETTA (1:5) binder samples with a thickness of about 0.2 cm were obtained. The resulting samples were dried in a vacuum oven at 120 °C for 1 h. The raw-PVdF binder sample was prepared by a solution casting method. The materials for the fabrication and the resulting electrolyte uptake (EU) of each raw-binder are summarized in Table 2.

### FE-SEM image of composite electrodes

**Figure 6:**
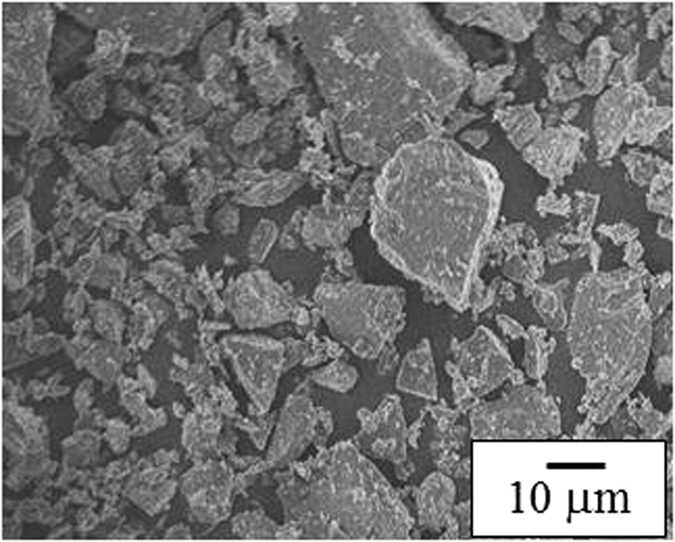
FE-SEM image of the Si particles used in this study.

**Figure 7:**
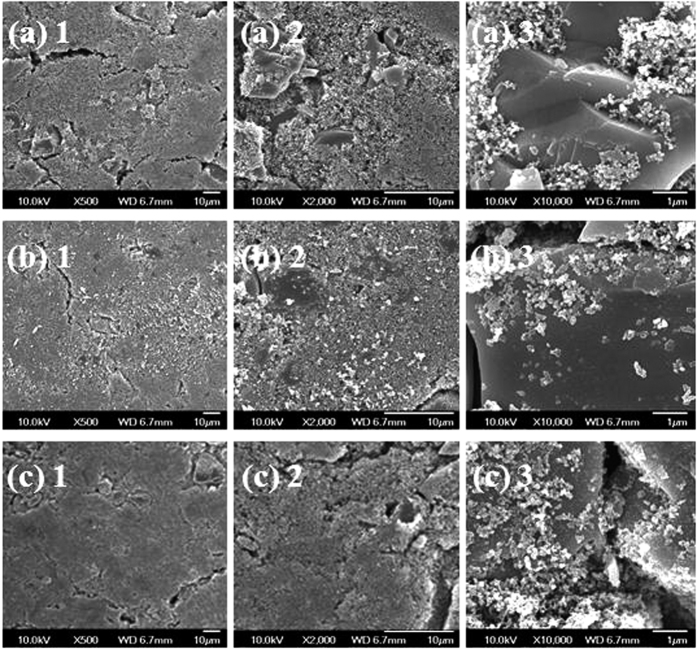
(**a**) FE-SEM images of the PVdF-composite, (**b**) the PPETTA-composite, and (**c**) the PVdF/PPETTA-composite (1:1).

Figure 6 shows a FE-SEM image of the Si used in this study. The morphology of the Si in this case was highly irregular. The average particle size was approximately 10 um. Figure 7 shows FE-SEM images of the PVdF-composite, the PPETTA-composite and the PVdF/PPETTA-composite (1:1). As shown in Fig. 7(a) 1 and 2, the surface of the PVdF-composite cracked frequently. In addition, the CB particles in this case were highly aggregated (Fig. 7(a) 3). However, there were far fewer, surface cracks in the PPETTA-composite (Fig. 7(b) 1) and the PVdF/PPETTA-composite (1:1) (Fig. 7(c) 1) compared to the PVdF-composite. The CB particles in these case (Fig. 7(b) 3 and (c) 3) were very well dispersed. Judging from this, the cross-link networks of the PPETTA as a binder played a role in reinforcing the binding strength between the electrode particles within the composite electrodes^5^,^6^. This most likely occurs because the CB particle inter-distances for the PPETTA-composite and the PVdF/PPETTA-composite (1:1) expanded as the PETTA monomer chains were extended through a curing process^3^,^4^,^5^.

### Electrolyte uptake (EU) of the binders

**Figure 8:**
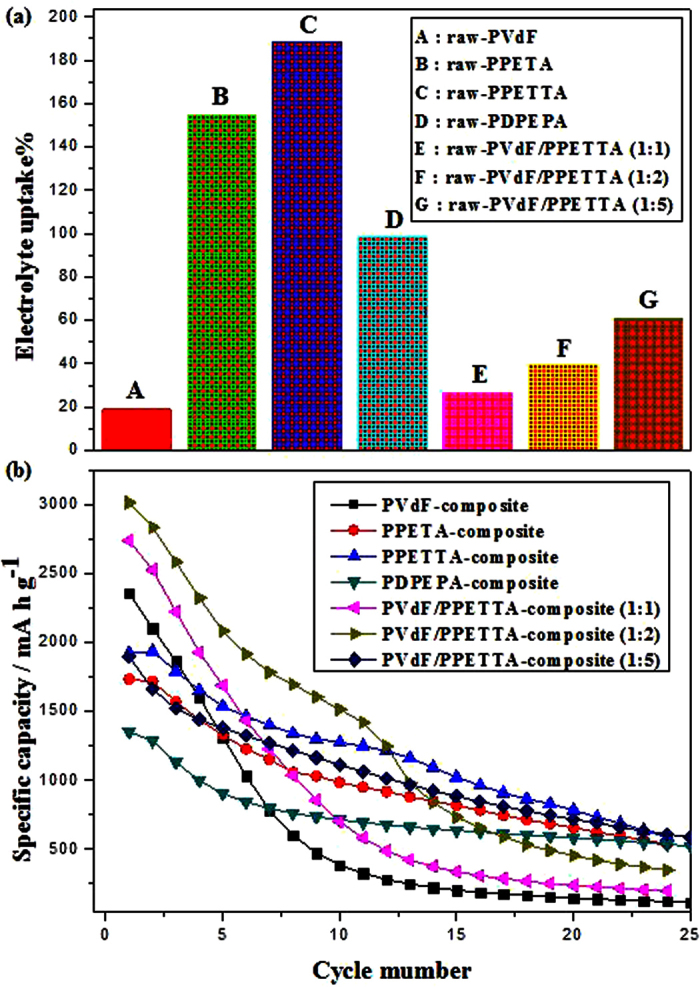
(**a**) EUs of the raw PVdF, PPETA, PPETTA, PDPEPA and PVdF/PPETTA binders and (**b**) cycle performance levels of the PVdF-composite, PPETA-composite, PPETTA-composite, PDPEPA-composite and PVdF/PPETTA-composites.

Figure 8(a) shows the electrolyte uptake of the PVdF, PPETA, PPETTA, PDPEPA and PVdF/PPETTA binders. Interestingly, the electrolyte uptake levels for the PPETA, PPETTA and PDPEPA samples were approximately 154.4%, 188.5% and 98.2%, close to 8, 10 and 5 fold greater than that of the PVdF sample (approximately 18.9%) despite the fact that they are cross-linking polymers. The PPETTA sample had the best electrolyte uptake. Because the electrolyte solution is absorbed only into the hydrophobic segments^4^,^5^,^7^, we consider that the electrolyte uptake levels of the PPETA and PDPEPA samples containing the hydroxyl groups (–OH, hydrophilic segments) at the side chain were decreased compared to that of the PPETTA sample^4^,^5^,^8^. The corresponding electrolyte uptake levels of the PVdF/PPETTA (1:1), PVdF/PPETTA (1:2) and PVdF/PPETTA (1:5) binders were approximately 26.7%, 39.4% and 60.5%.

### Electrochemical properties of the composite electrodes

Figure 8(b) shows the cycle performance of the PVdF-composite, the PPETA-composite, the PPETTA-composite, the PDPEPA-composite and the PVdF/PPETTA-composites. The discharge of the PVdF-composite amounted to 2359 mAh g^−1^, and it decreased by approximately 34% compared to the theoretical specific capacity of Si. In this case, the charge dropped sharply after one cycle, with only close to 8% (about 202 mAh g^−1^) of the discharge maintained after 15 cycles. Because the Li-ions migrate through the electrolyte-sorbed binder matrix^5^,^7^, it is expected that the poor electrolyte uptake of the PVdF decreased the discharge by reducing the number of Li-ionic carriers in the binder matrix. This occurred because the formation of an unstable solid electrolyte interphase (SEI) layer causes uninterrupted electrolyte solution degradation at the surface of the Si during the cycling process^9^,^10^,^11^,^12^,^13^,^14^,^15^,^16^. The abrupt reduction of the charge that occurred after one cycle can be attributed to the considerable volume expansion and the collapse of Si within the composite electrode^1^,^2^,^15^,^16^. As shown in Fig. 5, the surface cracks on the morphology of the PVdF-composite were much worse than on the other samples after 10 cycles. The PVdF-composite was nearly detached from the current collector.

On the other hand, the respective discharge amounts for the PPETA-composite, PPETTA-composite and PDPEPA-composite were approximately 1733 mAh g^−1^, 1921 mAh g^−1^ and 1352 mAh g^−1^, with corresponding decreases of 51%, 46 and 62% compared to the theoretical specific capacity of Si. All cases showed much less discharge than the PVdF-composite. Despite the high electrolyte uptake of the cross-linked polymer binders, this most likely occurred because the excessive cross-linking networks of the PPETA, PPETTA and PDPEPA increased the amount of Li trapping by blocking the Li-ion channels in the binder matrix^3^,^5^,^6^,^17^. In that the cross-linked density increases with an increase in the number of carbon-carbon double bonds in a functional monomer^6^,^8^,^13^,^17^, we expect that the discharge of the PDPEPA-composite was decreased compared to those of the PPETA-composite and the PPETTA-composite. Nevertheless, the charge in these three corresponding cases remained at approximately 47% (about 818 mAh g^−1^) of the discharge, at approximately 53% (about 1022 mAh g^−1^) of the discharge, and at approximately 47% (about 636 mAh g^−1^) of the discharge for 15 cycles. These values are nearly, 4, 5 and 3 times higher than that of the PVdF-composite. Moreover, the PPETA-composite, PPETTA-composite and PDPEPA-composite showed much better surface morphologies than the PVdF-composite after 10 cycles (Fig. 5). Accordingly, we believe that the cross-linked polymer networks of PPETA, PPETTA and PDPEPA as binders played an important role through volume variation of Si and in maintaining the binding strength within the composite electrodes during the cycling process^18^,^19^,^20^,^21^,^22^. This could occur because the robust cross-linking binder system reduced the deformation of SEI layers and the mechanical stress of crystalline Li_15_Si_4_ within the composite electrodes^9^,^10^,^11^,^12^,^13^,^14^,^15^,^16^.

The discharge amounts of the PVdF/PPETTA-composite (1:1), the PVdF/PPETTA-composite (1:2), and the PVdF/PPETTA-composite (1:5) were approximately 2739 mAh g^−1^, 3013 mAh g^−1^ and 1897 mAh g^−1^, respectively, showing decreases of approximately 22%, 15 and 47% compared to the theoretical specific capacity of Si. Specifically, the discharge amounts of the PVdF/PPETTA-composite (1:1) and the PVdF/PPETTA-composite (1:2) improved remarkably by approximately 380 mAh g^−1^ and 654 mAh g^−1^ respectively, compared to that of the PVdF-composite. These outcomes can be attributed to the fact that the numbers of Li traps of the PVdF/PPETTA binder (1:1) and the PVdF/PPETTA binder (1:2) decreased as the volume of the cross-linked PPETTA domain in the binder matrix was reduced^6^,^18^,^19^. The charge in these respective cases remained at approximately 12% (about 337 mAh g^−1^) of the discharge, at about 24% (about 733 mAh g^−1^) of the discharge, and at nearly 46% (about 884 mAh g^−1^) of the discharge for 15 cycles, increasing with an increase in the content of the cross-linked PPETTA in the blending binder matrix. The entire charge pattern for the PVdF/PPETTA-composite (1:5) was similar to that of the PPETTA-composite during the cycling process. The charge patterns of the PVdF-composite, PPETA-composite, PPETTA-composite, PDPEPA-composite and PVdF/PPETTA-composites during the cycling process are shown in Table 3.

According to work by Dong *et al*.^11^ the discharge amount of micro-Si negative electrodes for LIBs with sodium carboxymethyl cellulose (Na-CMC) as a binder was approximately 2150 mAh g^−1^, showing a decrease of 40% compared to the theoretical specific capacity of Si. The charge in this case was approximately 1770 mAh g^−1^ after one cycle. Park *et al*.^7^ also showed that (poly)vinyl alcohol (PVA) as a binder maintained excellent cyclic retention of Si/graphites due to its numerous hydroxyl groups. The discharge amount for their Si/graphites negative electrode was approximately 1500 mAh g^−1^. Koo *et al*.^6^ reported that the discharge amounts of Si composite electrodes with cured PAA-CMC, PAA and PVdF binders were approximately 2850 mAh g^−1^, 2200 mAh g^−1^ and 300 mAh g^−1^ respectively, at a current density of 300 mA g^−1^. As mentioned earlier, these studies depended only on a linear-polymer as a binder. The lower electrochemical performances reported in those studies may be due to the weak linear-polymeric binding system used or the poor electrolyte uptake levels of the binders within the Si negative electrodes in comparison to our study.

In conclusion, despite the fact that the charge of the PPETA-composite, the PPETTA-composite and the PDPEPA-composite as investigated here increased sharply during the cycling process, the discharge in these cases dropped significantly compared to that of the PVdF-composite. These outcomes were improved considerably by blending a linear-polymer binder and a cross-linked polymer binder through a curing process. These results could stem from the precise manipulation of the electrolyte uptake and cross-linking level of the binder within the composite electrodes.

## Methods

### Materials

Si was purchased from Aldrich and used as received (powder, −325 mesh, 99% trace metals basis). PETA (molecular formula: C_14_H_18_O_7_, Mw: 298.24, CAS number: 3524-68-3, density: 1.18 g/mL at 25 °C (lit.), Refractive index: *n*20/D 1.483 (lit.), Flash point: >230 °F), PETTA (molecular formula: C_17_H_20_O_8_, Mw: 352.34, CAS number: 4986-89-4, density: 1.19 g/mL at 25 °C (lit.), Refractive index: *n*20/D 1.487 (lit.), Flash point: >230 °F), DPEPA (molecular formula: C_25_H_32_O_12_, Mw: 524.52, CAS number: 60506-81-2, density: 1.155 g/mL at 25 °C (lit.), Refractive index: *n*20/D 1.49 (lit.), Flash point: >230 °F), CB (Denka black) and NMP (molecular formula: C_5_N_9_NO_2_, Mw: 115.13, CAS number: 41194-00-7) were also purchased from Aldrich and used as received. AIBN (molecular formula: C_8_H_12_N4, Mw: 164.21, CAS number: 78-67-1) was purchased from DEEJUNG CHEMICALS & METALS CO., LTD.

### Coin half-cell measurements

Coin half cells (CR2032) were manufactured in a dry glove box with ethylene carbonate (EC)/ethyl methyl carbonate (EMC) (3:7 vol. ratio) as an electrolyte containing 1.3 M of LiPF_6_ and Celgard^®^ commercial trilayer PP/PE/PP separators. Lithium metal was used as a counter electrode. The galvanostatic cycle was carried out in a voltage range of 0~2.0 V with a current density of 100 mA/g (WBCS 3000 cycler, Wonatech Co., Korea).

### EU measurement

The EU of the prepared raw-binders was determined by measuring the change in the weight between the wet and dry binder. The raw-binders were soaked in an EC/EMC (3:7 vol. ratio) electrolyte solution containing 1.3 M of LiPF_6_ at room temperature for 48 h. The external electrolyte was wiped off, and the binders were weighed. The electrolyte uptake amounts of the binders were obtained by the following equation:


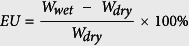


Here, *W*_dry_ and *W*_wet_ are the weight of the dried and the electrolyte-sorbed binder, respectively.

### Morphology measurement

Dispersed electrode particle images of the prepared electrodes were confirmed by a field emission scanning electron microscope (FE-SEM, Hitachi Co. Japan).

## Additional Information

**How to cite this article**: Jang, S.-Y. and Han, S.-H. Fabrication of Si negative electrodes for Li-ion batteries (LIBs) using cross-linked polymer binders. *Sci. Rep.* **6**, 38050; doi: 10.1038/srep38050 (2016).

**Publisher's note:** Springer Nature remains neutral with regard to jurisdictional claims in published maps and institutional affiliations.

## Figures and Tables

**Table 1:** Materials for the fabrication of the PVdF-composite, PPETA-composite, PPETTA-composite, PDPEPA-composite and PVdF/PPETTA-composites.

Sample	Si	CB	PVdF	PETA	PETTA	DPEPA	AIBN	NMP	Curing
PVdF-composite	0.60 g	0.25 g	0.15 g	—	—	—	—	3.0 g	NO
PPETA-composite	0.60 g	0.25 g	—	0.15 g	—	—	0.005 g	3.0 g	YES
PPETTA-composite	0.60 g	0.25 g	—	—	0.15 g	—	0.005 g	3.0 g	YES
PDPEPA-composite	0.60 g	0.25 g	—	—	—	0.15 g	0.005 g	3.0 g	YES
PVdF/PPETTA-composite (1:1)	0.60 g	0.25 g	0.075 g	—	0.075 g	—	0.005 g	3.0 g	YES
PVdF/PPETTA-composite (1:2)	0.60 g	0.25 g	0.05 g	—	0.10 g	—	0.005 g	3.0 g	YES
PVdF/PPETTA-composite (1:5)	0.60 g	0.25 g	0.025 g	—	0.125 g	—	0.005 g	3.0 g	YES

**Table 2:** Materials for the fabrication and the resulting EU of each raw binder.

Sample	PVdF	PETA	PETTA	DPEPA	AIBN	NMP	Curing	Casting	EU
raw-PVdF binder	1.5 g	—		—	—	15 g	NO	YES	18.9%
raw-PPETA binder	—	1.5 g	—	—	0.005 g	15 g	YES	NO	154.4%
raw-PPETTA binder	—	—	1.5 g	—	0.005 g	15 g	YES	NO	188.5%
raw-PDPEPA binder	—	—	—	1.5 g	0.005 g	15 g	YES	NO	98.2%
raw-PVdF/PPETTA (1:1) binder	0.75 g	—	0.75 g	—	0.005 g	15 g	YES	NO	26.7%
raw-PVdF/PPETTA (1:2) binder	0.5 g	—	1.0 g	—	0.005 g	15 g	YES	NO	39.4%
raw-PVdF/PPETTA (1:5) binder	0.25 g	—	1.25 g	—	0.005 g	15 g	YES	NO	60.5%

**Table 3:** Charge pattern of the PVdF-composite, PPETA-composite, PPETTA-composite, PDPEPA-composite and PVdF/PPETTA-composite during the cycling process.

Sample	1 cycle	5 cycle	10 cycle	15 cycle	20 cycle	25 cycle
PVdF-composite	100%	55%	16%	8%	6%	4%
	(2359 mAhg ^−1^)	(1312 mAhg^−1^)	(385 mAhg ^−1^)	(202 mAhg ^−1^)	(145 mAhg ^−1^)	(116 mAhg^−1^)
PPETA-composite	100%	76%	56%	47%	37%	31%
	(1733 mAhg ^−1^)	(1328 mAhg ^−1^)	(985 mAhg ^−1^)	(818 mAhg^−1^)	(655 mAhg ^−1^)	(539 mAhg ^−1^)
PPETTA-composite	100%	80%	66%	53%	40%	30%
	(1921 mAhg^−1^)	(1537 mAhg^−1^)	(1275 mAhg ^−1^)	(1022 mAhg ^−1^)	(784 mAhg ^−1^)	(589 mAhg ^−1^)
PDPEPA-composite	100%	66%	52%	47%	43%	38%
	(1352 mAhg ^−1^)	(905 mAhg^−1^)	(712 mAhg^−1^)	(636 mAhg ^−1^)	(583 mAhg ^−1^)	(522 mAhg ^−1^)
PVdF/PPETTA-composite (1:1)	100%	61%	25%	12%	8%	7%
	(2739 mAhg ^−1^)	(1688 mAhg ^−1^)	(701 mAhg^−1^)	(337 mAhg^−1^)	(237 mAhg ^−1^)	(197 mAhg^−1^)
PVdF/PPETTA-composite (1:2)	100%	69%	50%	24%	15%	11%
	(3013 mAhg ^−1^)	(2084 mAhg ^−1^)	(1515 mAhg ^−1^)	(733 mAhg^−1^)	(454 mAhg ^−1^)	(348 mAhg ^−1^)
PVdF/PPETTA-composite (1:5)	100%	72%	58%	46%	37%	32%
	(1897 mAhg^−1^)	(1377 mAhg^−1^)	(1112 mAhg ^−1^)	(884 mAhg^−1^)	(716 mAhg^−1^)	(609 mAhg ^−1^)

**Figure 1:**
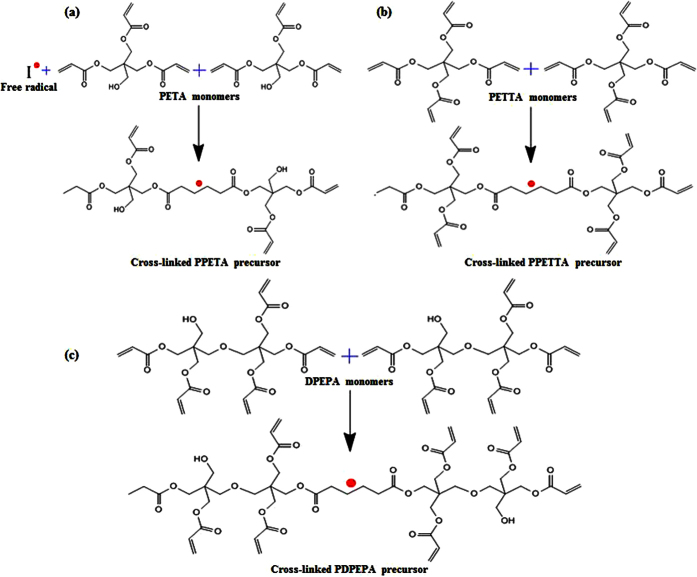
Cross-linking routes of the PETA (**a**), PETTA (**b**) and DPEPA (**c**) multifunctional monomers.
